# Image review on mobile devices for suspected stroke patients: Evaluation of the mRay software solution

**DOI:** 10.1371/journal.pone.0219051

**Published:** 2019-06-28

**Authors:** Alex Brehm, Volker Maus, Eya Khadhraoui, Marios-Nikos Psychogios

**Affiliations:** 1 Department of Neuroradiology, University Medical Center Goettingen, Goettingen, Germany; 2 Department of Neuroradiology, Clinic of Radiology & Nuclear Medicine, University Hospital Basel, Basel, Switzerland; 3 Department of Radiology, Neuroradiology and Nuclear Medicine, Ruhr University Hospital Bochum, Knappschaftskrankenhaus Bochum, Bochum, Germany; University of Ioannina School of Medicine, GREECE

## Abstract

**Purpose:**

Software solutions such as mRay allow review of radiological images on handheld devices. We investigated if the quality is adequate for evaluating CT scans of patients with suspected stroke.

**Methods:**

50 patients (Median age 80 years, 28 females) were retrospectively selected. All patients had undergone multidetector CT angiography ± perfusion and presented with clinical signs of acute stroke. Out of the 50 patients, 19 had large-vessel occlusion (LVO), 5 had intracranial hemorrhage (ICH), 10 had severe intracranial stenosis of at least one major vessel, 2 had intracranial tumor and 15 had no or an unrelated pathology. One experienced neuroradiologist and one resident scored the anonymized pictures separately on two handheld devices (iPhone 7 Plus, MED-TAB) equipped with mRay Software and on a PACS workstation. Each case was reviewed on all three devices with a break in-between of at least 12 weeks. The scoring on the traditional workstation was compared with the two handheld devices, regarding detection of early ischemic signs, LVOs, CBV/CBF-mismatch, ICHs and severe stenosis. Both raters were asked to rate the diagnostic quality of both handheld devices regarding detection of LVOs, ICHs, early ischemic signs and overall.

**Results:**

All LVOs, intracranial tumors and ICHs were detected on both mobile devices. There was no significant difference in the rating of CCT and CBF ASPECTS between all three devices, while the sensitivity for detecting a CBF/CBV-mismatch was above 80% on both devices. Both raters assessed the diagnostic quality to be sufficient on both mobile devices to base treatment decisions on.

**Conclusion:**

Software solutions such as mRay for handheld devices provide adequate diagnostic quality for the review of CT scans of suspected stroke patients.

## Introduction

Since the first digital picture archival and communication system (PACS) has been introduced in 1995, the gold-standard for reviewing diagnostic images moved from film to computer-based display workstations.[1] This allows the diagnostic radiologist to view the images immediately and simultaneously on multiple workstations. Furthermore it gives the user tools to magnify, rotate and calculate measures on images. However one major drawback of computer based workstation is the lack of portability. Especially for a senior on call, this represents a severe limitation since the physician is confined to a location with a workstation in reach. This problem can be addressed with a mobile solution. In 2011 the first manuscript on such a mobile solution was published.[2] Since then, rapid evolvement of digital networks, smartphone and handhelds occurred and allowed next generation mobile solutions, which feature most functions a PACS has to offer. mRay for handheld devices is such a solution, which is a certified medical device (Class IIb). It enables the physician to review medical images in the digital imaging and communications in medicine (DICOM) format on a number of handheld devices such as smartphones and tablets. We sought to determine if the aforementioned software used on handheld devices such as an iPhone or the MED-TAB provide sufficient image quality to base clinical decisions in case of a suspected stroke.

## Methods

The patient database of a comprehensive stroke center was screened for patients, which presented with a suspected stroke in the anamnesis in the year 2017 (Keywords: “NIHSS”,”Schlaganfall”) and received at least a multidetector CT (MDCT) scan. The first fifty consecutive patients were selected for further review. All images were acquired using a SOMATOM Definition AS+ system (Siemens healthcare GmbH, Forchheim, Germany). The native CT was reconstructed in 4 mm multiplanar reconstructions on a commercially available workstation (syngo X Workplace, Siemens). The CT angiography was reviewed in 0.75 mm thick slices and on reconstructed maximal intense projections with a slide thickness of 10 mm. CT perfusion maps were reconstructed out of the 5 mm thick CT perfusion data set. Data were prospectively collected and documented in an Institutional Review Board-approved database, which was approved under the number 13/7/15An by the ethics committee of the University Medicine Gottingen The need for an additional formal application or separate consent regarding the inclusion in our retrospective study was waived. The study was conducted in accordance with the Declaration of Helsinki.

One experienced senior in neuroradiology (VM>5 years of experience) and one resident (EK>1 year of experience) scored the anonymized scans independently on two handheld devices and on a GE PACS workstation. Each case was reviewed on all three devices with a break in-between of at least twelve weeks, to minimize recall effects. In all three review cycles all three devices were used and the cases were grouped into three groups consisting of 17, 17 and 16 cases. Each group was reviewed on another device in each cycle and the scans were randomized before each cycle. For each case the scoring physician had access to all available pictures. We did not specify the ambient light conditions to allow review in a natural environment, however both physicians were asked to review the images at a location with an ambient light intensity of under 100 lux. Both physicians were asked to rate the scans regarding the native and CBV Alberta stroke program early CT score (ASPECTS), the detection of ICH, LVO, CBV/CBF-mismatch, relevant stenosis of the ICA or the VA or other pathologies (i.e. tumors, unrelated pathologies such as pulmonic infarction and so on). Furthermore the physicians were asked to evaluate the diagnostic quality of all devices on a five point ordinal scale (0 = no diagnostic value, 1 = poor diagnostic value, 2 = adequate diagnostic value, 3 = good diagnostic value, 4 = excellent diagnostic value) regarding exclusion of an ICH, detection of an LVO, delineation of early ischemic signs and overall safety of the diagnosis.

The portable system consisted either of a MED-TAB (PLUM Medical Solutions GmbH, Rostock, Germany) or an iPhone 7 plus (Apple Inc, Cupertino, CA), client image viewing software (mRay, mbits imaging GmbH, Heidelberg, Germany) and a data server. The MED-TAB had 16 GB of flash memory and a 13.3-inch diagonal screen with 1920 x 1080 pixels (luminance > 250 cd/m^2^). It is DICOM Part 14 greyscale standard display function certified. The iPhone 7 plus had 128 GB of flash memory and a 5.5-inch diagonal screen with 1920 x 1080 pixels (luminance = 625 cd/m^2^). Both portable devices ran the mRay mobile client program, which handled the user input and communication with the data server and display of the transmitted images. The software allows all standard operations of GE PACS i.e. translation, rotation, linking of different series, measuring distances and zooming. Communication between the handheld devices and the data server occurred over a secured wireless (WIFI 802.11 g) or LTE/mobile network. Gold-standard diagnosis occurred on a standard radiology workstation equipped with GE PACS connected to two medical-grad 21 inch liquid crystal displays (RX250, EIZO). Both displays had a resolution of 1000 x 1600 pixels and a luminance of 400 cd/m^2^.

Descriptive statistics and contingency tables were used for statistical evaluation. Sensitive and specificity was calculated for dichotomized values. Ordinal variables were compared with the matched-pair Wilcoxon test. Intraobserver agreement was evaluated with weighted κ statistics, with a value above κ_w_  =  0.6, representing substantial agreement[3]. Analyses were performed with the MedCalc Statistical Software version 18 (MedCalc Software bvba, Ostend, Belgium; http://www.medcalc.org; 2018).

## Results

All fifty patients (Median age 80 years, 28 females) were included in this study. The majority of the patients received a standard CT scan plus angiography and perfusion (n = 39), while 9 patients received a standard CT scan plus angiography and 2 patients received only a standard CT scan. Out of the 50 patients 19 had a large-vessel occlusion (LVO), 5 had an intracranial hemorrhage (ICH) (3 intraparenchymal bleedings and 2 subarachnoid bleedings), 10 had a severe stenosis of at least 1 major vessel (9 A. carotis interna (ICA); 3 of them NASCET > 70%, 3 A. vertebralis (VA); 1 of them NASCET > 70%), 2 patients had an intracranial tumor and 15 patients had no or an unrelated pathology.

Both raters identified all 5 ICHs correctly on both devices (see Table 1). The 19 LVOs were detected as well on both devices. Regarding the LVO location there was slight disagreement in 3 cases (see Table 2), however the affected territory and side was correct in all cases. The senior scored the native ASPECTS with a median of 10 (IQR 10–10) on all three devices. There was no significant difference between the scoring on the workstation and the Med-TAB (p = 0.641) and the iPhone 7 plus (p = 0.423). No significant difference was detected for the resident as well (p = 0.250 (Med-TAB) and p = 0.162 (iPhone 7 plus)). The median was 10 (IQR 10–10) on all three devices. Both the senior and the resident rater scored the CBV ASPECTS with a median of 10 and an IQR of 9–10 on all three devices. There were no significant differences regarding the scoring of the CBV ASPECTS on all three devices (see Table 3).

**Table 1:** Sensitivity and specificity for ICH, LVO, CBV/CBF-missmatch and Stenosis of the ICA/A. vertebralis.

Variable	Rater	Prevalence (GE PACS)	Med-TAB		iPhone 7 plus	
			Sensitivity Value in % (95% CI)	Specificity Value in % (95% CI)	Sensitivity Value in % (95% CI)	Specificity Value in % (95% CI)
Intracranial hemorrhage	Senior	5	100 (47.8–100)	100 (92.1–100)	100 (47.8–100)	100 (92.1–100)
	Resident	5	100 (47.8–100)	100 (92.1–100)	100 (47.8–100)	100 (92.1–100)
Large vessel occlusion	Senior	19	100 (82.4–100)	100 (88.1–100)	100 (82.4–100)	100 (88.1–100)
	Resident	19	100 (82.4–100)	100 (88.1–100)	100 (82.4–100)	100 (88.1–100)
CBV/CBF-mismatch	Senior	17	84.2 (56.6–96.2)	91.3 (72.0–98.9)	88.2 (63.6–98.5)	90.9 (70.8–98.9)
	Resident	20	85.0 (62.1–96.8)	84.2 (60.4–96.6)	85 (62.1–96.8)	83.3 (58.6–96.4)
Intracranial Tumor	Senior	2	100 (15.8–100)	100 (92.6–100)	100 (15.8–100)	100 (92.6–100)
	Resident	2	100 (15.8–100)	100 (92.6–100)	100 (15.8–100)	100 (92.6–100)
Stenosis of the ICA	Senior	10	66.7 (29.9–92.5)	100 (91.0–100)	55.6 (21.2–86.3)	94.9 (82.7–99.4)
	Resident	9	50.0 (18.7–81.3)	89.2 (74.6–97.0)	60.0 (26.2–87.8)	94.3 (80.8–99.3)
Stenosis of the A. vertebralis	Senior	3	33.3 (0.8–90.6)	100 (92.1–100)	66.7 (9.4–99.2)	97.8 (88.2–99.9)
	Resident	4	50 (6.8–93.2)	93.0 (80.9–98.5)	50 (6.8–93.2)	88.4 (74.9–96.1)

CBF, cerebral blood flow, CBV cerebral blood volume, ICA internal carotid artery

**Table 2:** Vessel location of the large-vessel occlusion for each device.

Case	Rater	GE PACS	MED-TAB	iPhone 7 Plus
1	Senior	M2 Segment	M2 Segment	M2 Segment
	Resident	M2 Segment	M2 Segment	M2 Segment
3	Senior	M1 Segment	M1 Segment	M1 Segment
	Resident	M1 Segment	M1 Segment	M1 Segment
4	Senior	M1 Segment	M1 Segment	M1 Segment
	Resident	M1 Segment	M1 Segment	M1 Segment
5	Senior	M1 Segment	M1 Segment	M1 Segment
	Resident	M1 Segment	M1 Segment	M1 Segment
6	Senior	Basilar Artery	Basilar Artery	Basilar Artery
	Resident	Basilar Artery	Basilar Artery	Basilar Artery
7	Senior	M2 Segment	M2 Segment	M2 Segment
	Resident	M2 Segment	M2 Segment	M2 Segment
9	Senior	**M1 Segment**	**ICA T**	**ICA T**
	Resident	ICA T	ICA T	ICA T
10	Senior	Basilar Artery	Basilar Artery	Basilar Artery
	Resident	Basilar Artery	Basilar Artery	Basilar Artery
12	Senior	ICA T	ICA T	ICA T
	Resident	ICA T	ICA T	ICA T
20	Senior	M1 Segment	M1 Segment	M1 Segment
	Resident	M1 Segment	M1 Segment	M1 Segment
21	Senior	Basilar Artery	Basilar Artery	Basilar Artery
	Resident	Basilar Artery	Basilar Artery	Basilar Artery
22	Senior	ICA T	ICA T	ICA T
	Resident	ICA T	ICA T	ICA T
23	Senior	M1 Segment	M1 Segment	M1 Segment
	Resident	M1 Segment	M1 Segment	M1 Segment
28	Senior	**M3 Segment**	**M3 Segment**	**M2 Segment**
	Resident	M2 Segment	M2 Segment	M2 Segment
39	Senior	**M1 Segment**	**M1 Segment**	**ICA T**
	Resident	M1 Segment	M1 Segment	M1 Segment
41	Senior	ICA T	ICA T	ICA T
	Resident	ICA T	ICA T	ICA T
44	Senior	ICA T	ICA T	ICA T
	Resident	ICA T	ICA T	ICA T
47	Senior	M3 Segment	M3 Segment	M3 Segment
	Resident	M3 Segment	M3 Segment	M3 Segment
49	Senior	M1 Segment	M1 Segment	M1 Segment
	Resident	M1 Segment	M1 Segment	M1 Segment

ICA T, intracranial carotid artery terminus

**Table 3:** CCT and CBV ASPECTS on the MED-TAB/iPhone plus 7 and GE PACS.

Variable	Rater	Modality	Score Median (IQR)	Wilcoxon p Value (PACS gold-standard)
CCT ASPECTS	Senior	PACS	10 (10–10)	-
		MED-TAB	10 (10–10)	0.641
		iPhone	10 (10–10)	0.423
	Resident	PACS	10 (10–10)	-
		MED-TAB	10 (10–10)	0.250
		iPhone	10 (10–10)	0.162
CBV ASPECTS	Senior	PACS	10 (9–10)	-
		MED-TAB	10 (9–10)	0.240
		iPhone	10 (9–10)	1
	Resident	PACS	10 (9–10)	-
		MED-TAB	10 (9–10)	0.492
		iPhone	10 (9–10)	1

ASPECTS, Alberta stroke program early computer tomography score, CBV, cerebral blood volume CCT, cranial computer tomography, IQR interquartile range

These results are reflected as well in the substantial intrarater agreement for the ASPECTS (see Table 4) with 0.717 ± 0.110 between the MED-TAB and GE PACS and 0.709 ± 0.092 between the iPhone 7 plus and GE PACS for the senior and 0.787 ± 0.057 and 0.797 ± 0.056 for the resident. The interrater agreement on all three devices was substantial as well on all three devices. For the detailed results please refer to Table 5. The sensitivity for detecting a CBV/CBF-mismatch was 84.2% (95% CI 56.6–96.2%) on the MED-TAB and 88.2% (95% CI 63.6–98.5%) on the iPhone 7 plus in case of the senior and 85.0% (95% CI 62.1–96.8%) and 85.0% (95% CI 62.1% - 96.8%) in case of the resident. The corresponding specificity was 91.3% (95% CI: 72.0–98.9%) for the MED-TAB and 90.9% (95% CI: 70.8–98.9%) for the iPhone 7 Plus in case of the senior and 84.2% (95% CI: 62.1–96.8%) and 83.3% (95% CI: 58.6–96.4%) for the resident. The CBV ASPECT showed substantial intrarater agreement between the MED TAB and GE PACS with 0.695 ± 0.096 and between the iPhone 7 plus and GE PACS with 0.766 ± 0.088 for the senior. In case of the resident the intrarater agreement was also substantial with a weighted kappa of 0.735 ± 0.097 and 0.740 ± 0.101. The 2 intracranial tumors were detected correctly on both devices by both raters.

**Table 4:** Intrarater agreement for CCT and CBV ASPECTS between MED-TAB/iPhone plus 7 and GE PACS.

Variable	Rater	CCT ASPECTS	CBV ASPECTS
Weighted Κ ± SE MED-TAB vs GE PACS	Senior	0.717 ± 0.110	0.695 ± 0.096
	Resident	0.787 ± 0.057	0.735 ± 0.097
Weighted Κ ± SE iPhone plus 7 vs GE PACS	Senior	0.709 ± 0.092	0.766 ± 0.088
	Resident	0.797 ± 0.056	0.740 ± 0.101

ASPECTS, Alberta stroke program early computer tomography score, CBV, cerebral blood volume CCT, cranial computer tomography

**Table 5:** Interrater agreement for CCT and CBV ASPECTS between the senior and the resident on all three devices.

Weighted Κ ± SE	CCT ASPECTS	CBV ASPECTS
GE PACS	0.722 ± 0.081	0.682 ± 0.107
MED-TAB	0.681 ± 0.086	0.797 ± 0.050
MED-TAB	0.657 ± 0.097	0.788 ± 0.070

ASPECTS, Alberta stroke program early computer tomography score, CBV, cerebral blood volume CCT, cranial computer tomography

The senior detected 12 severe stenosis (>50% after NASCET) of which 9 were in the ICA and 3 were in the VA. The sensitivity for detecting a severe stenosis in the ICA was 66.7% (95% CI: 29.9–92.5%) on the MED-TAB and 55.6% (95% CI: 21.2–86.3%) on the iPhone 7 Plus, while the specificity was 100% (95% CI: 91.0–100%) and 94.5% (95% CI: 82.7–99.4%) respectively. In case of a stenosis of the VA the sensitivity was 33.3% (95% CI: 0.8–90.6%)) on the MED-TAB and 66.7% (95% CI: 9.4–99.2%) on the iPhone 7 Plus, while the specificity was 100% (95% CI: 92.1–100%) and 97.8% (95% CI: 88.2–99.9%) respectively. The resident detected 14 severe stenosis (>50% after NASCET) of which 10 were in the ICA and 4 were in the VA. The sensitivity for detecting a severe stenosis in the ICA was 50% (95% CI 18.7–81.3%) on the MED-TAB and 60.0% (95% CI: 26.2–87.8%) on the iPhone 7 plus, while the specificity was 89.2% (95% CI: 74.6–97.0%) and 94.3% (95% CI: 80.8–99.3%) respectively. In case of a stenosis of the VA the sensitivity was 50% (95% CI: 6.8–93.2%) on the MED-TAB and 50% (95% CI: 6.8–93.2%) on the iPhone 7 Plus, while the specificity was 93.0% (95% CI: 80.9–98.5%) and 88.4% (95% CI: 74.9–96.1%) respectively.

Both raters felt safe to exclude an ICH (see Table 6) on both handheld devices and rated the diagnostic value to be at least adequate in 97.8% of the cases, while they rated it perfect in at least 82.4% of the cases. There was no difference between the diagnostic value of the GE PACS and the MED-TAB (p = 0.875 senior and p = 0.833 resident) or the iPhone 7 plus (p = 1 senior and p = 0.622 resident). Regarding the safe detection of an LVO the senior rated the mobile devices in all cases to be sufficient for a diagnosis, while the resident rated the MED-TAB in 97.9% (p = 0.170) to be sufficient for a diagnosis and the iPhone plus 7 in 95.8% (p = 0.230) to be sufficient for a diagnosis. Early ischemic signs could be safely diagnosed in 94% of the cases on both mobile devices, while this was possible in 98% of the cases in GE PACS according to the senior, however this difference was not statistical significant (p = 0.946 and p = 0.112). The resident felt safe to diagnose them in 92% of the cases in GE PACS, in 96% on the MED-TAB (p = 0.699) and in 95% on the iPhone 7 plus (p = 0.893). Overall the senior rated the two mobile devices and GE PACS to be sufficient for a diagnosis in all cases, while he rated the MED-TAB to be of perfect diagnostic value in 68%, the iPhone 7 plus in 66% and the GE PACS in 76% of the cases. However this difference was not statistical significant (p = 0.217 and p = 0.068). The resident rated the MED-TAB to be sufficient for a diagnosis in 96%, the iPhone 7 plus in 94% compared to GE PACS in 98%, this difference was not statistical significant as well (p = 0.181 and p = 0.956)

**Table 6:** Qualitative rating of diagnostic value of the three devices.

Variable	Rater	Modality	No diagnostic value n (%)	Little diagnostic value n (%)	Adequate diagnostic value n (%)	Good diagnostic value n (%)	Perfect diagnostic value n(%)	Wilcoxon p Value (PACS gold-standard)
Safety exclusion of ICH	Senior	PACS	0 (0)	0 (0)	1 (2.2)	1 (2.2)	43 (95.6)	-
		MED-TAB	0 (0)	0 (0)	1 (2.2)	2 (4.4)	42 (93.4)	0.875
		iPhone	0 (0)	0 (0)	1 (2.2)	1 (2.2)	43 (95.6)	1
	Resident	PACS	0 (0)	1 (2.2)	1 (2.2)	5 (11)	38 (84.8)	-
		MED-TAB	0 (0)	0 (0)	2 (4.4)	5 (11)	38 (84.8)	0.833
		iPhone	0 (0)	1 (2.2)	3 (6.6)	4 (8.8)	37 (82.4)	0.622
Safety detection of an LVO	Senior	PACS	0 (0)	0 (0)	1 (2.1)	5 (10.4)	42 (87.5)	-
		MED-TAB	0 (0)	0 (0)	0 (0)	7 (14.6)	41 (85.4)	1
		iPhone	0 (0)	0 (0)	2 (4.2)	10 (20.8)	36 (75.0)	0.067
	Resident	PACS	1 (2.1)	4 (8.3)	4 (8.3)	18 (37.5)	21 (43.7)	-
		MED-TAB	0 (0)	1 (2.1)	7 (14.6)	15 (31.2)	25 (52.1)	0.170
		iPhone	0 (0)	2 (4.2)	8 (16.7)	12 (25)	26 (54.2)	0.230
Safety detection of early ischemic signs	Senior	PACS	1 (2)	0 (0)	9 (18)	16 (32)	24 (48)	-
		MED-TAB	3 (6)	0 (0)	5 (10)	19 (38)	23 (46)	0.946
		iPhone	0 (0)	3 (6)	12 (24)	17 (34)	18 (36)	0.112
	Resident	PACS	1 (2)	3 (6)	1 (2)	15 (30)	30 (60)	-
		MED-TAB	0 (0)	2 (4)	4 (8)	12 (24)	32 (64)	0.699
		iPhone	1 (2)	2 (4)	5 (10)	11 (22)	31 (62)	0.893
Overall diagnostic value	Senior	PACS	0 (0)	0 (0)	0 (0)	12 (24)	38 (76)	-
		MED-TAB	0 (0)	0 (0)	2 (4)	14 (28)	34 (68)	0.217
		iPhone	0 (0)	0 (0)	3 (6)	14 (28)	33 (66)	0.068
	Resident	PACS	0 (0)	1 (2)	8 (16)	25 (50)	16 (32)	-
		MED-TAB	0 (0)	2 (4)	5 (10)	19 (38)	24 (48)	0.181
		iPhone	0 (0)	3 (6)	6 (12)	24 (48)	17 (34)	0.956

ICH, intracranial hemorrhage, LVO, large vessel occlusion

## Discussion

**Fig 1:**
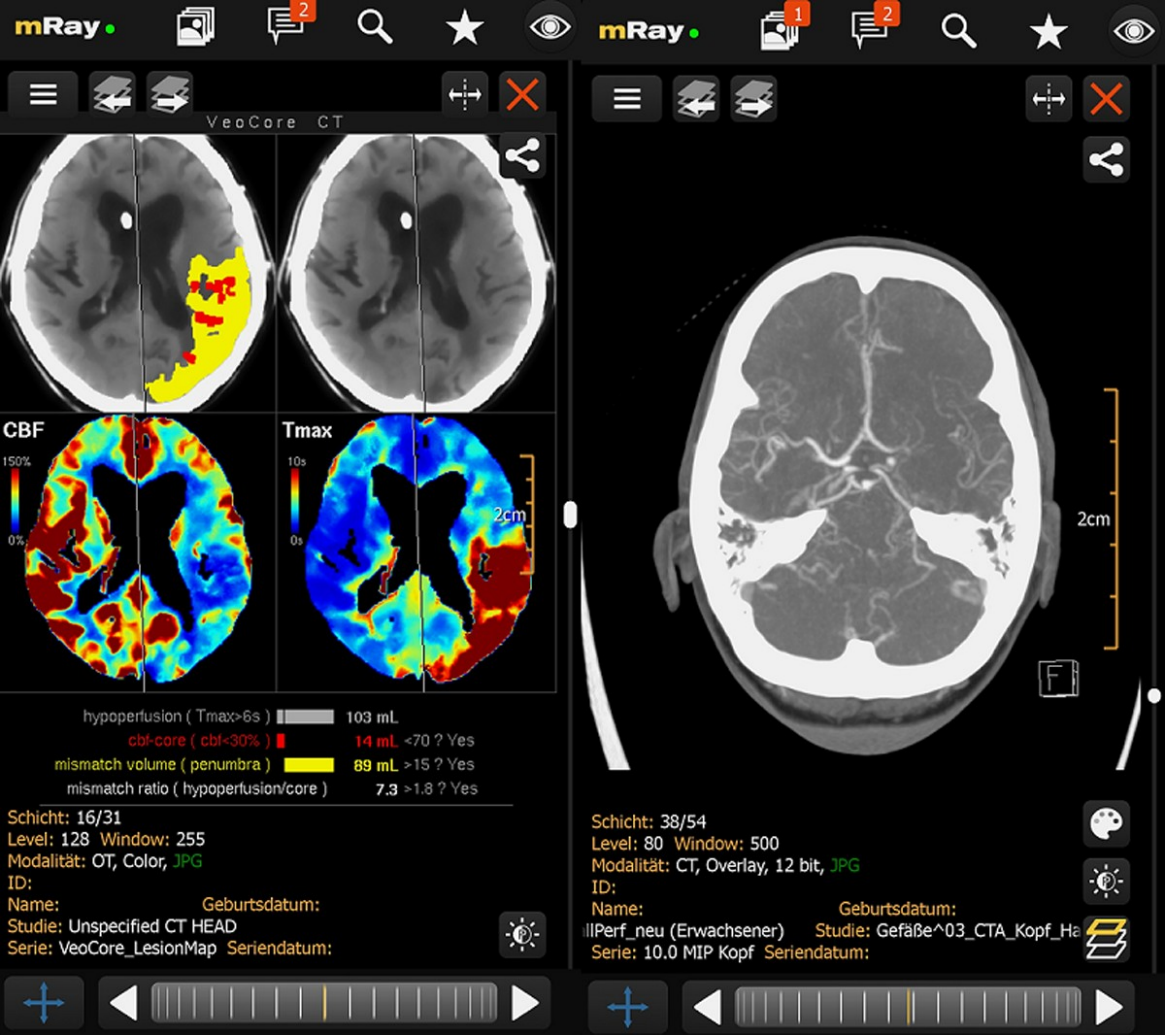
Veocore lesion map with core and mismatch and LVO on a CTA scan viewed with mRay.

This study shows, that mobile devices such as the MED-TAB or a commercially available smartphone of the newer generation in conjunction with a DICOM viewing software (in case of this study mRay) are sufficient for both a senior and a second year resident to safely rule out ICHs and detect LVOs. Regarding the detection of early ischemic signs all three devices seem to be diagnostically equivalent, since we were unable to detect any difference in the rating of the ASPECTS between all three devices for both raters. This is reflected as well by the substantial intrarater agreement, which parallels the results of papers which evaluated the ASPECTS on computer based workstations with certified displays[4,5]. This replicates findings from previous work done by Randhawa et al., who showed in a smaller case series that although in six out of 18 cases additional findings were reported after reviewing the cases again on a PACS workstation, only one of them contributed to the diagnosis[6]. Furthermore the rating of the CBV ASPECTS was comparable on all three devices as well. This enables the physician to make decisions regarding further care of stroke patients even on a mobile device. The ability to safely rule out ICHs, gives him the capacity to assist the treating neurologist in the decision to administer IV-tPA. Furthermore since the physician can detect LVOs and interpret CBV maps sufficiently on a mobile device, the physician is able to decide to perform a mechanical thrombectomy without the need of a conventional workstation. Therefor our results might impact the working environment, especially for the senior on call, since with raising numbers of suspected stroke patients and interventions his workload is increasing.[7] As our results indicate that the radiological scans of suspected stroke patients can be reviewed and assessed sufficiently on a mobile device, it gives the senior on-call the opportunity to be mobile while being on-call. Besides the opportunity to review images at any location, the mobile solution might lead to an acceleration of the decision-making process. Regarding the decision to perform thrombectomy, automated algorithms for the interpretation of CT perfusion data such as rapid[8] or veocore[9] (see Fig 1), which was already introduced into the mRAY software, while we performed this study, might increase diagnostic safety even further. The results of our study are in line with the experiences of our senior doctors, who are using mRAY on a mobile device and didn`t have to revoke their decisions regarding IV-tPA or mechanical thrombectomy in the last two years. Furthermore in urban areas 4G or at least 3.5G is readily available and provides data bandwidth of at least 7.2 Mbit/s, which is comparable to home network connections.

The detection rate of intracranial stenosis of the ICA and the VA was low on both mobile devices compared to the gold-standard, however 8 out of 12 stenosis had a NASCET rating between 50 and 69% and a large meta-study done by Wardlaw et al. showed similar rates (Sensitivity 67% (95% CI: 30–90%)) for such stenosis[10]. Furthermore the sample size was low with only 12 cases of severe stenosis in our sample. However we would still advocate reviewing the images later on a traditional workstation to identify minor findings such as stenosis or unrelated pathologies.

Although both reviewers felt safe assessing CT/CTA and CTP studies on a handheld device, there are advantages of a traditional workstation especially compared to smartphones. The most important disadvantage of the handheld device is, that on a smart phone the maximum number of parallel viewed series is 2 and on the MED-TAB it is 4, while on a traditional workstation at least 8 series can be comfortable viewed parallel. This advantage might have contributed to the markedly higher findings of intracranial stenosis on the workstation. Another advantage of the mobile device is the possibility of mobile communication through the mRay app, which allows the coordination of the physicians prior to the mechanical Thrombectomy.

The primary limitation of our study is its retrospective design, which might have led to a selection bias. Also recall effects cannot be ruled out completely, although they were minimized by randomization and the in-between time of at least twelve weeks. Furthermore we did not control for ambient light conditions, however Randhawa et al. showed that the impact of the ambient light seems to be negligible as long as the images are reviewed in any conditions which are comparable to indoor conditions[6].

### Conclusion

Our study shows that mobile devices can be safely used for image review of CT scans of suspected stroke patients and in case of an experienced rater treatment decisions can be based on such reviews. However for assuring, that additional findings are not missed reviewing the scans later on a traditional workstation should still be mandatory.

## Abbreviations

ICA: Arteria carotis interna
VA: Arteria vertebralis
ASPECTS: Alberta stroke program early CT score
CBF: Cerebral blood flow
CBV: Cerebral blood volume
CTA: Computer tomography angiography
CTP: Computer tomography perfusion
DICOM: Digital imaging and communications in medicine
ICH: Intracranial hemorrhage
IV-tPA: Intravenous tissue plasminogen activator
LVO: Large vessel occlusion
MDCT: Multidetector computer tomography
NASCET: North American Symptomatik Carotid Endarterectomy Trial
PACS: Picture archival and communication system
